# Ag/AgBr-oxygen enriched g-C_3_N_4_ for efficient photocatalytic degradation of trimethylamine

**DOI:** 10.1039/d4ra02395a

**Published:** 2024-04-29

**Authors:** Xinru Chen, Zeyu Duan, Feiyang He, Haiqiang Wang, Zhongbiao Wu

**Affiliations:** a Key Laboratory of Environment Remediation and Ecological Health, Ministry of Education, College of Environmental & Resources Science, Zhejiang University Hangzhou 310058 P.R. China haiqiangwang@zju.edu.cn; b Zhejiang Provincial Engineering Research Center of Industrial Boiler & Furnace Flue Gas Pollution Control Hangzhou 310058 P. R. China

## Abstract

In this study, Ag/AgBr–O-gCN samples with ternary Z-type heterojunctions were prepared by *in situ* photoreduction using water as the reducing agent for generating Ag/AgBr active species and oxygen doping. The experimental results indicated that Ag/AgBr–O-gCN degraded trimethylamine by nearly 100% in half an hour and maintained 90% of its original activity after five cycles. The kinetic constant of Ag/AgBr–O-gCN was excellent at 0.0928 min^−1^, 3.8 times that of gCN, 2.3 times that of Ag/AgBr-gCN, and 1.9 times that of O-gCN. Unlike Ag/AgBr-gCN photoreduced by methanol, gCN was used as an electron donor in the aqueous solution during the photoreduction process, and oxidation sites between the gCN skeleton and Ag/AgBr were formed for constructing the heterojunction system. The Z-type heterojunction system was established by introducing a suitable size of Ag nanoparticles as the recombination center to keep indirect contact between gCN and AgBr. This effectively reduced the electron–hole recombination rate and caused activity enhancement. This study offers a novel idea for the construction of a ternary heterojunction.

## Abstract 2

In this study, Ag/AgBr–O-gCN samples with ternary Z-type heterojunctions were prepared by *in situ* photoreduction using water as the reducing agent for generating Ag/AgBr active species and oxygen doping.

## Graphical abstract

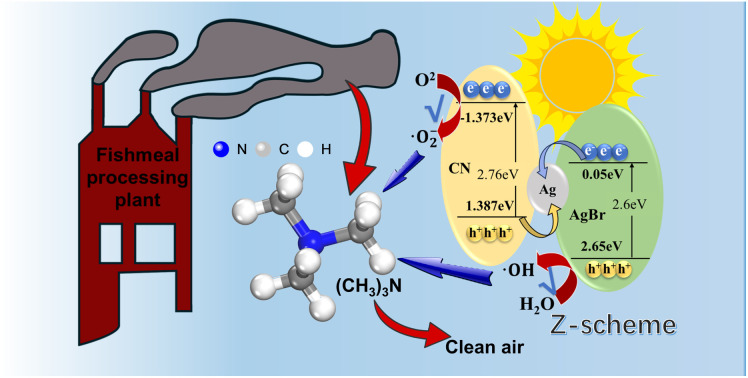

## 1 Introduction

Malodorous gases stimulate the olfactory organs, cause unhappiness, and damage the living environment.^1^ Trimethylamine (TMA) is a typical nitrogen-containing malodorous gas, which has the characteristics of extremely low olfactory threshold and various pollution sources. The molecular formula of TMA is (CH_3_)_3_N, obtained by replacing the H on NH_3_ with CH_3_, and it is generally released by the decomposition of trimethylamine *N*-oxide (TMAO).^2–5^

Researchers have adopted many methods to eliminate TMA at low concentrations, including biological treatments,^6^ adsorption,^7^ and nonthermal plasma.^8^ However, these traditional methods have drawbacks, such as poor stability, secondary pollution, and high operation and maintenance costs, which may result in resource wastage.^9^ In 2021, Yan^2^ used a simple hydrothermal method to deposit Co metal on the surface of MoS_2_, and used external bias voltage in the photoelectric chemical system to separate photogenerated e^−^/h^+^ pairs, improve the efficiency of photoelectric catalytic degradation of TMA. The limitation of Yan is that the gas-phase pollutants are still converted to the liquid phase for degradation, and the conversion process will have problems such as low gas–liquid mass transfer efficiency. Therefore, it is imperative to develop an efficient and stable photocatalyst that can complete the photocatalytic degradation of TMA under gas-phase conditions.

Recently, photocatalytic degradation has attracted increasing attention. As a green and clean treatment method, it features low cost and high efficiency for treating ultralow concentrations of organic pollutants.^10,11^ At present, the photocatalytic degradation of TMA is seldom studied, and mainly focuses on the regulation of reaction conditions such as working conditions, and the photocatalyst was generally TiO_2_. Assadi^12^ carried out photocatalytic degradation experiments on TMA and found that the efficiency of TiO_2_ is 25%. The large band gap of TiO_2_ is proved to be the factor restricting its application in photocatalytic degradation. Graphite carbon nitride (g-C_3_N_4_, referred to as gCN in this paper) has been widely reported for its good chemical stability, suitable electronic structure, and appropriate bandgap for sunlight utilization.^13^ The bandgap of gCN is generally 2.7 eV, with the conduction band (abbreviated as CB) at −1.1 eV and the valence band (abbreviated as VB) at 1.6 eV, which is suitable for most photocatalytic reactions.^14^

However, owing to its high recombination rate of photoexcited charge carriers in its 2D nanosheet plane, and low specific surface area, original gCN exhibits unsatisfactory photocatalytic performance.^15^ To improve photocatalytic performance, several modification methods have been developed, including defect adjustment, heteroatom doping, and morphology control.^16,17^ Non-metallic element doping is always considered an effective method for the modification of gCN. In particular, the heteroatom O, being more electronegative than the C and N that gCN already possessed,^18^ tends to be doped into the skeleton. Therefore, the negative charges accumulated on the O atoms to form electron-rich centers,^19^ which could promote carrier separation. Unfortunately, oxygen-enriched reagents, such as H_2_O_2_ (ref. 20) and oxalic acid dihydrate,^19^ are inevitably used in the synthesis of the O-reinforced gCN reported in most studies, but it is often questioned because of their cost and danger. If the heteroatom O can be doped into the gCN skeleton under mild conditions, this safety issue can be effectively addressed.

AgBr was proved to be a widely used photocatalyst and when Ag nanoparticles were introduced into the system, as a common noble metal cocatalyst, the SPR effect produced by Ag could promote the local electromagnetic field, then promptly generated electrons and holes in AgBr. Meanwhile, owing to that the photo-generated e^−^ on AgBr could be rapidly removed to Ag as the electron-withdrawing group, the barrier was formed to inhibit the charge recombination process, which is called Schottky Barrier.^21–23^ Based on its basic physicochemical properties, Ag/AgBr is expected to be more effective for the generation and transfer of electrons than traditional semiconductor-based photocatalysts.

Recently, ternary Z-scheme heterojunctions have shown great potential in photocatalysis and have been widely applied in areas listed as follows: water splitting,^24^ the purification of wastewater, such as tetracycline hydrochloride,^25^ and the degradation of VOCs, such as ethylene.^22^ Photodeposition is a common heterostructure construction method, and sacrificial agents, such as methanol, are used as reductants.^25^ In this study, we innovatively replaced methanol with water as a sacrificial agent, which made the gCN skeleton oxidized during the photoreduction process. We were looking forward to realizing the co-doping of Ag/AgBr as well as heteroatom O, and the construction of a ternary Z-type heterojunction in a one-step photoreduction process.

Ag/AgBr–O-gCN was prepared as a ternary heterojunction photocatalyst by the deposition of Ag/AgBr active species and the doping of O heteroatoms on g-C_3_N_4_ using an *in situ* photoreduction method and introduced for the photocatalytic degradation of TMA. The crystal structure, light trapping, and active species of Ag/AgBr–O-gCN were comprehensively analysed, and finally proposed an activity improvement mechanism for the Z-type heterostructure based on the C–O bond. This study led to a new doping method for oxygen-enriched g-C_3_N_4_, which combined heteroatom doping with heterostructure construction in a one-step photoreduction process and offered a new reference template for the photocatalytic degradation of nitrogen-containing malodorous gas pollutants.

## 2 Experimental

### 2.1 Preparation of Ag/AgBr–O-gCN catalysts

The synthesis of preparation of gCN was using a simple pyrolysis method of urea by crucible. The weight of urea is 20.0 g. The heating rate was 10 °C min^−1^ and was kept at 550 °C for 2 hours. The resulting powder is yellow. O-gCN was synthesized using the same method except that the amount of urea was changed to 10 g and 10 ml water was added into the crucible. The obtained O-gCN was also light yellow, but whiter than gCN.

**Fig. 1:**
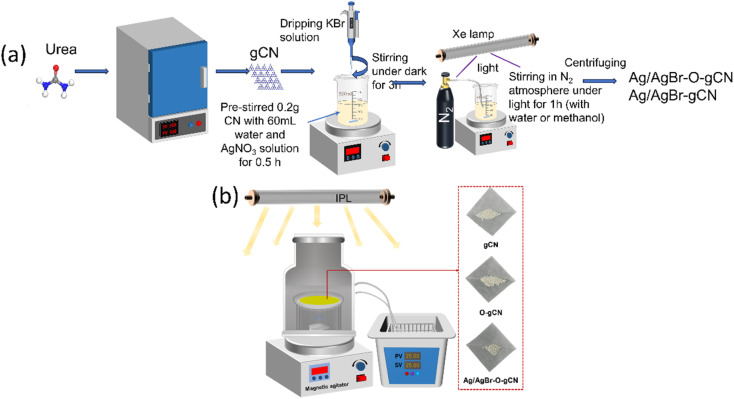
(a) A brief schematic diagram for the synthesis to prepare Ag/AgBr–O-gCN and Ag/AgBr-gCN. (b) The schematic diagram of the reaction device and the physical diagram of the sample.

The Ag/AgBr–O-gCN photocatalyst was prepared using an one-step *in situ* coprecipitation and photoreduction-assisted methods. Relevant details have been shown in Fig. 1. Solution A was obtained by dispersing 0.2 g gCN in 60 ml water or methanol, stirring continuously for 30 minutes. A different volume of AgNO_3_ (0.1 mol L^−1^) named solution B was added in A. Then the same volume of KBr (0.1 mol L^−1^) was dripped into A. Keep it stirred for 3 hours in dark. After this, the solution was irradiated with an Xe lamp in an N_2_ atmosphere for 1 h, and the sample was collected under centrifugation. The obtained grey-green precipitate was alternately washed three times and dried in an oven. The sample using methanol as a sacrificial agent during photoreduction was named Ag/AgBr-gCN. The sample using water as a sacrificial agent during photoreduction was named Ag/AgBr–O-gCN, and the initial Ag concentration of the general sample is 8%.

### 2.2 Catalytic performance tests

Photocatalytic degradation of the catalyst was tested in a quartz glass reactor with ambient temperature controlled at 25 °C. The round reactor was about 2.5 L, and a magnet was used for stirring to keep the concentration of pollutants in the tank consistent from top to bottom. The used light source was Intense Pulsed light (IPL).

For each experiment, put 50 mg photocatalyst on a round glass (*D* = 10 cm) and turn it into a film with the help of ethanol. After drying, place it at the bottom of the reactor. Then, TMA saturated steam was injected until the TMA concentration in the tank was 50 ppm. At intervals in the experiment, the gas sample was extracted from the tank by the 1 ml syringe and injected into the Gas Chromatography (GC, FULI9790, China) to measure the concentration of TMA quantitatively. The degradation rate of TMA was obtained by *E*_1_, where the original concentration was named TMA_0_ and TMA_t_ represented the concentration at the moment t.
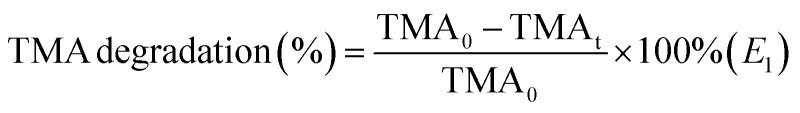


### 2.3 Characterizations

X-ray Diffraction (XRD) patterns were detected to analyse the crystal phases and structures (Rigaku SmartLab SE with Cu Kα radiation (*λ* = 0.15418 nm)). The scanning speed is 10° per min from 5 to 90°. Using software such as Jade and Origin to determine the position of the peak and generate the graph. The surface functional groups of the samples were detected by Fourier transform infrared (FTIR, Nicolet iS20, Thermo Scientific, America) spectra. The instrument used for the N_2_ adsorption and desorption experiments was a Beijing Jingwei Gaobo JW-BK 132F adsorption instrument, and according to Brunauer–Emmett–Teller (BET) and Barret–Joyner–Halender (BJH) models, the specific surface area, pore volume and pore size of the samples can be obtained. Scanning electron microscopy (Hitachi, SU8010) and transmission electron microscopy (FEl Tecnai G2 F20 S-TWIN) images were acquired to reveal the morphology and microstructures of the samples. The X-ray photoelectron spectroscopy (XPS) spectra were acquired by a Thermo Scientific K-Alpha (USA) instrument with an Al Kα microfocus monochrome source. For full-spectrum scanning, the general energy is 100 eV, and for Narrow-spectrum scanning is 30–50 eV. Binding energy correction was based on surface pollution C 1s (284.8 eV) as the standard for correction. UV-vis spectrophotometer diffuse reflectance spectra (DRS) of all the catalysts were measured using BaSO_4_ as a reference (Shimadzu, UV-2600, range from 200 to 800 nm). PL spectra were obtained with Edinburgh FLS1000 using a 390 nm laser as the excitation source. Photoelectrochemical tests on the electrochemical workstation (CHI660E) are performed including Electrochemical impedance spectra (EIS) and photocurrent. The photocurrent values were measured under dark and illumination conditions in turn, and each cycle was 100 s. The light source was a 300 W Xe lamp. Impedance was measured under dark conditions. Using an In-doped SnO_2_ (ITO) substrate (1 cm × 1 cm) coated with catalyst powders, the working electrode was prepared. Platinum wire and Ag/AgCl were separately employed as counter electrodes and reference electrodes. The aqueous Na_2_SO_4_ solution (0.20 M, pH = 6.8) was used as the electrolyte. Electron spin resonance (ESR) signals were examined (JES-FA200, Japan) under full spectrum light provided by a 300 W Xe lamp. The capture agent used to detect ˙OH− and ˙O_2_^−^ is 5,5-Dimethy-1-pyrroline *N*-oxide (DMPO), and for photo-generated electrons and holes it was 2,2,6,6-Tetramethyl-1-oxylpiperidine (TEMPO).

## 3 Results and discussion

### 3.1 Crystal form and structure

**Fig. 2:**
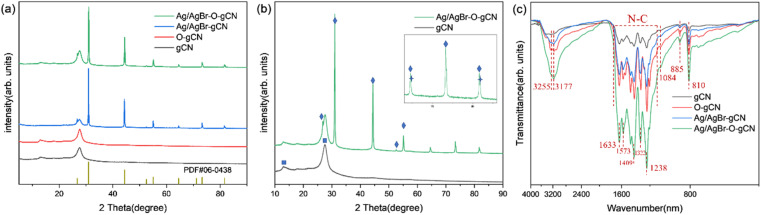
(a) and (b) XRD results of gCN, O-gCN, Ag/AgBr-gCN, and Ag/AgBr–O-gCN (square, diamond, and star symbols represent carbon nitride, AgBr, and Ag simple substances respectively). (c) FTIR patterns of gCN, O-gCN, Ag/AgBr-gCN, and Ag/AgBr–O-gCN.

The composition and crystal phases were researched by XRD analysis, and Fig. 2 showed the diffraction spectra of gCN, O-gCN, Ag/AgBr-gCN, and Ag/AgBr–O-gCN. The characteristic peaks of g-C_3_N_4_, Ag, and AgBr can be observed in Fig. 2a, indicating the existence of these three components. The gCN possessed a regular arrangement on an in-plane structure united by a three-striped azine ring and a common interlayer spacing between its layers,^26^ owing to its characteristic peaks. One is at 13.1°, attributed to the crystal plane (100), and the other was the interlayer-stacking peak at 27.5°, considered to be (002). The general XRD peaks of AgBr appeared at 26.7, 31.0, 44.3, 55.1, 64.6, 73.2, and 81.6°, noted as square, corresponding to the crystal planes (111), (200), (220), (222), (400), (420), and (422) of AgBr, respectively (JCPDS: 06-0438). Meanwhile, although the diffraction peaks of Ag appeared to be weak, they existed at 44.5, 64.5, and 81.9°, corresponding to the crystal planes (200), (220), and (222) of Ag (JCPDS: 04-0783). The reason for Ag's weak peaks was that the content of Ag in the catalyst was really low. Simultaneously, the result also represented the uniform distribution of Ag particles.^4,22^ The overlap with the main peaks of AgBr was also a reason that made it hard to distinguish.

The similar diffraction peaks of Ag/AgBr–O-gCN and Ag/AgBr-gCN in Fig. 2a prove that photoreduction without methanol is also effective in converting Ag^+^ to Ag^0^ nanoparticles. Compared to Ag/AgBr–O-gCN, the diffraction peak of gCN in Ag/AgBr-gCN was weaker because of the poor crystallinity of gCN, which might be another reason why the degradation effect of Ag/AgBr-gCN was not as good as that of Ag/AgBr–O-gCN.^12^ As for the others, the two samples' diffraction peaks of Ag and AgBr were almost identical.

The FTIR absorption peaks of the catalysts were almost the same in Fig. 2c, and the triazine ring was represented by the respiratory vibration peak at 810 cm^−1^. The C–N heterocycle stretching vibration peaks at 1200–1650 cm^−1^ and –NH at 3000–3400 cm^−1^ were observed clearly in Fig. 2c.^27^ It was found that after Ag/AgBr modification, the intensity of the triazine ring and C–N heterocycle were enhanced, which was attributed to the strong force between the Ag/AgBr and gCN, indirectly indicating that Ag/AgBr and gCN were closely bonded rather than simply physically attached.^25^ The residual –NH_*x*_ vibration peaks at 3177 cm^−1^ and 3255 cm^−1^ on the surface of gCN became stronger.^28^ The tensile vibration of C–O at 1084 cm^−1^ as well as the N–O group peak at 980 cm^−1^ of Ag/AgBr–O-gCN and O-gCN indicated that C and N were oxidized during the photodeposition process.^19,20,29,30^ The FTIR spectra showed that the gCN skeleton was oxidized to a certain extent when Ag/AgBr was introduced into the photoreduction process with water as a reducing agent.

**Fig. 3:**
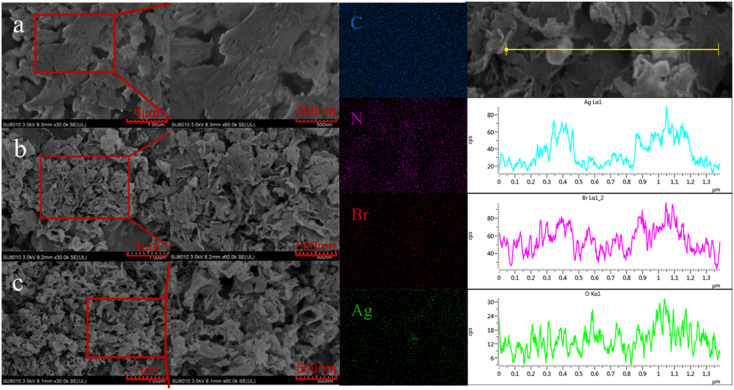
(a–c) SEM of gCN, O-gCN, Ag/AgBr–O-gCN. The EDS spectrum belongs to Ag/AgBr–O-gCN.

To further determine the microstructure of Ag/AgBr–O-gCN composites in detail, the materials were characterized by SEM. Compared with the original sample, Ag/AgBr–O-gCN nanoparticles had smaller particles and mesopores than the original sample. The EDS results showed that there were bright Ag/AgBr particles in the dark field, placing more bright spots belonging to Ag and Br. This result proved Ag/AgBr particles were successfully deposited on the surface of gCN by the photodeposition process. In the EDS diagram on the right side of Fig. 3, the enrichment of the O element appeared on the gCN skeleton, which may be related to the growth of Ag particles and AgBr species on the gCN skeleton. This is also evidence that the O element widely exists on the gCN skeleton.

**Fig. 4:**
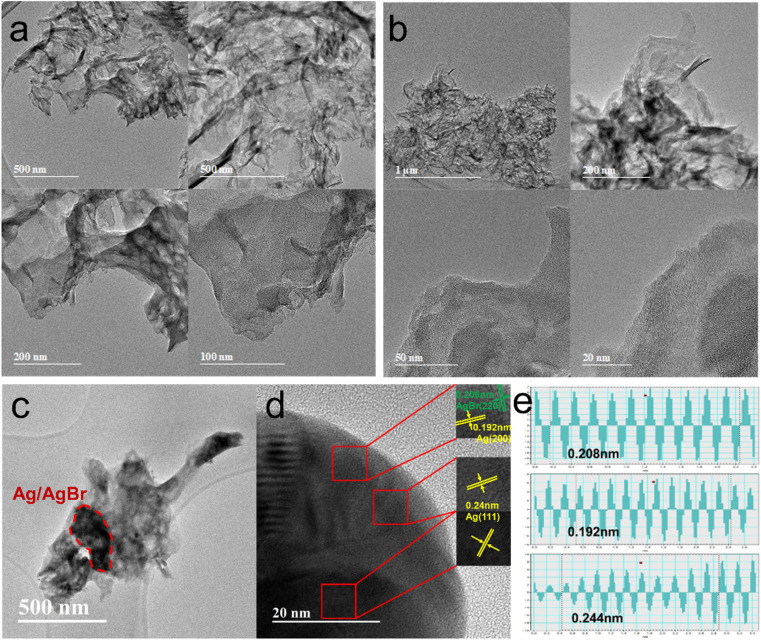
TEM images of gCN (a), O-gCN (b) and TEM images showed different lattice stripes of Ag/AgBr–O-gCN (c–e).

The general layered structure of the gCN and O-gCN tested by TEM were shown in Fig. 4a and b. It was observed that the O-gCN nanoparticles had smaller particles and more pores than the original gCN sample, which was more helpful to electron transfer. In the TEM diagram of Fig. 4c, there is an image of a typical contact between gCN and AgBr. The black area emphasized by the red line is the place where AgBr attaches, and darker Ag particles grow on its surface. In Fig. 4d, the lattice fringes of Ag and AgBr were observed, measured, and proved to be 0.2996 nm for AgBr (200) and 0.2334 nm for Ag (111), respectively.^22^ This phenomenon indicated that reducing Ag accumulated and formed nanoparticles on the sample's surface, as well as AgBr particles. It can be observed that the crystallinity of gCN is not very good, and no obvious lattice stripes are observed. The measurement process of lattice fringes was reflected in Fig. 4e, proving that 0.208 nm, 0.192 nm, and 0.244 nm are for AgBr (220), Ag (200), and Ag (111), respectively.^31^ The aforementioned TEM results showed that the Z-type heterojunction structure was formed by gCN, Ag, and AgBr and it was convenient for photogenerated charges to transfer. As an electron storage pool in close contact with gCN and AgBr, Ag prolonged the electron transfer path to a certain extent and also offered electron traps to decrease the recombination of e^−^–h^+.^^32^

**Fig. 5:**
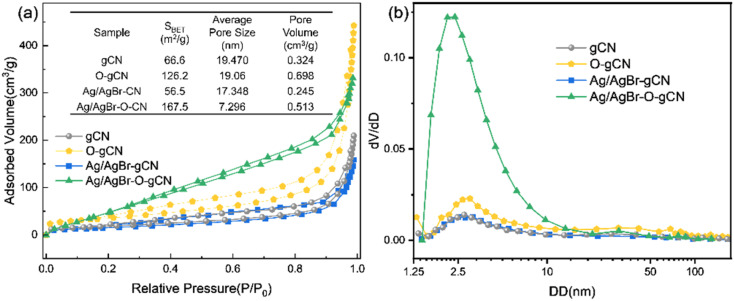
(a) Adsorption–desorption curves of gCN, O-gCN, Ag/AgBr-gCN, and Ag/AgBr–O-gCN (the detailed message about specific surface area, average pore size, and pore volume of samples was presented on interpolation charts). (b) The differential distribution curve of pore volume-pore size.

Fig. 5a shows the N_2_ adsorption–desorption curves for gCN, O-gCN, Ag/AgBr-gCN, and Ag/AgBr–O-gCN samples. All samples exhibited the characteristics of the H3 hysteresis loop without an obvious adsorption saturation platform, indicating the samples' irregular pores. Moreover, through BET-BJH model transformation, *S*_BET_ and the size as well as volume of average pore of Ag/AgBr–O-gCN were more competitive than that of gCN, which were shown in the embedded table in Fig. 5. The *S*_BET_ of Ag/AgBr–O-gCN was as high as 167.5 m^2^ g^−1^, which was much higher than that of gCN (66.6 m^2^ g^−1^). Fig. 5b shows that the micropore content is the highest in several samples, among which Ag/AgBr–O-gCN is the most notable.

### 3.2 Chemical composition and surface element analysis

**Fig. 6:**
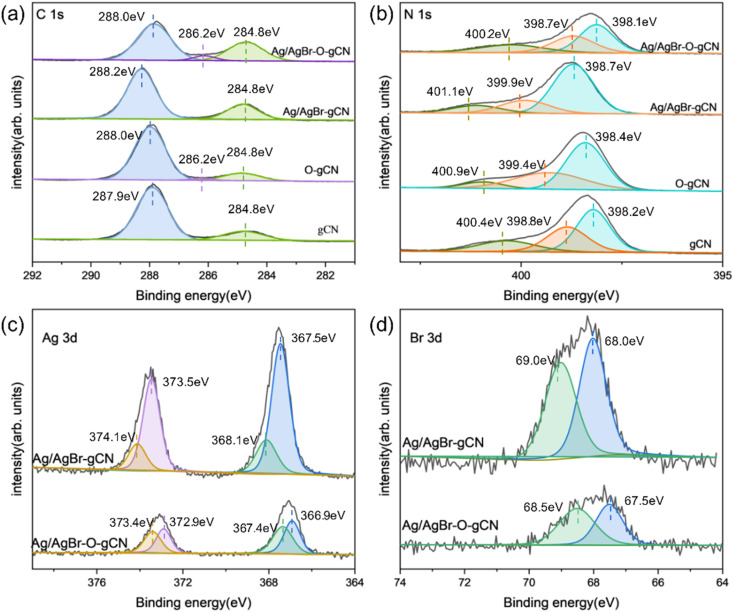
(a–d) C 1s, N 1s, Ag 3d, Br 3d high-resolution XPS of gCN, Ag/AgBr–O-gCN, and Ag/AgBr-gCN.

XPS analysis (Fig. 6) revealed the chemical state of catalysts throughout the photoreduction deposition treatment. Two peaks of C1s at 284.8 eV and 288.0 eV were found in Fig. 6a.^15^ The first peak was the sp^2^ C–C bonds and the second peak corresponded to the aromatic skeleton rings of gCN as C (N–C=N). Notably, a novel peak appeared and became stronger at 286.2 eV for O-gCN and Ag/AgBr–O-gCN, which corresponded to the bonding of C–O–C, implying that the C in gCN was partially oxidized, consistent with the results of FTIR.^19,20,30^

The N 1s XPS signal of gCN showed three peaks at 398.1, 398.7, and 400.2 eV, respectively, which come from the sp^2^ hybrid nitrogen, tertiary nitrogen N–(C)_3_, and N–H bond in C–N=C group.^15^ The peak position shifted to a lower binding energy after water photoreduction, representing the electron loss of the N skeleton. However, for Ag/AgBr-gCN, the gCN skeleton did not show signs of oxidation because methanol was used as the sacrificial agent.

Fig. 6c shows the spectroscopy of Ag 3d. Peaks at four different positions were obtained by further fitting, indicating the typical Ag 3d peak with a splitting of the 3d doublet spacing 6.0 eV.^33^ The peaks at 366.9 eV and 372.9 eV corresponded to Ag^+^ 3d_5/2_ and Ag^+^ 3d_3/2_, respectively. and 367.4 eV and 373.4 eV belonged to Ag^0^ 3d_5/2_ and Ag^0^ 3d_3/2_, indicating the existence of pure Ag particles.^20,33^ The binding energies of Br 3d_5/2_ and Br 3d_3/2_ were 67.5 and 68.5 eV, indicating that the negative valence of Br existed in the system.^21,23,25,31,32^

### 3.3 TMA photocatalytic degradation performance

**Fig. 7:**
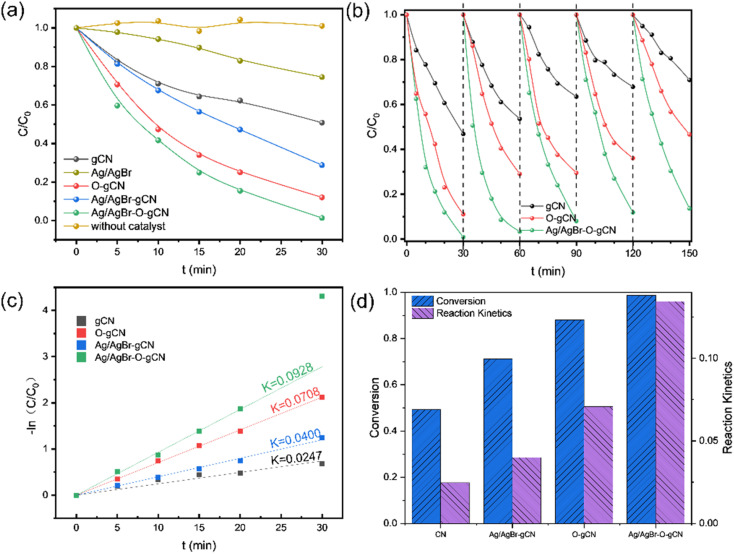
(a) Photocatalytic performance in 30 min of Ag/AgBr–O-gCN, Ag/AgBr-gCN, O-gCN, gCN, and Ag/AgBr; (b) results of repeated and recycling tests of gCN, O-gCN, Ag/AgBr–O-gCN; (c) reaction kinetic constant of Ag/AgBr–O-gCN, Ag/AgBr-gCN, O-gCN and gCN, (d) summary of the above results.

Fig. 7a shows the photocatalytic degradation activity of TMA for different samples. Experimental results indicated that Ag/AgBr had the worst performance, followed by gCN. Ag/AgBr–O-gCN revealed excellent photocatalytic degradation performance for TMA, with a kinetic constant as excellent as 0.0928 min^−1^, which is 3.8 times that of gCN, 2.3 times that of Ag/AgBr-gCN, and 1.3 times that of O-gCN. These results strongly suggest that the ternary Z-type heterojunction and the enriched C–O bonds were beneficial for photocatalytic activity. The regeneration ability of the prepared catalyst was studied by a continuous cycle experiment, with results shown in Fig. 7b. Ag/AgBr–O-gCN showed the best stability, maintaining 90% photocatalytic degradation efficiency after being reused five times.

**Fig. 8:**
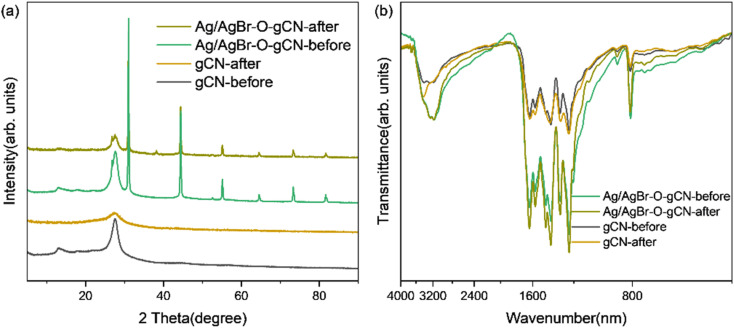
(a) XRD and (b) FTIR spectra of Ag/AgBr–O-gCN before and after the reaction.

**Fig. 9:**
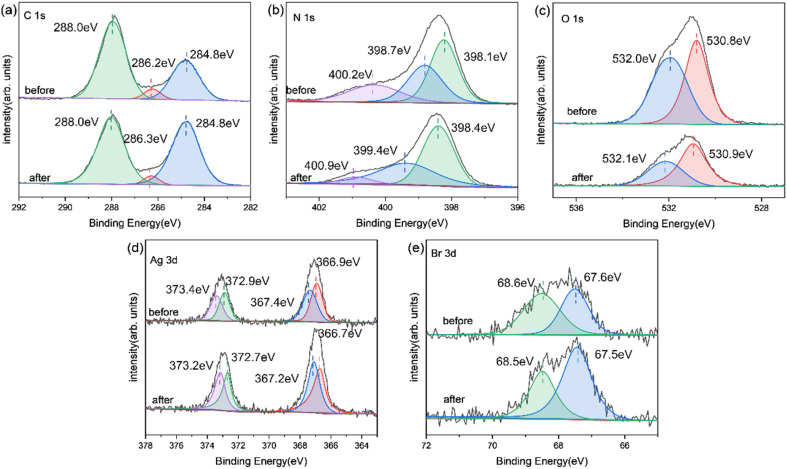
XPS image of Ag/AgBr–O-gCN before and after the reaction: (a) C 1s; (b) N 1s; (c) O 1s; (d) Ag 3d; (e) Br 3d.

Fig. 8 and 9 show the XRD, FTIR, and XPS results before and after the reaction. No differences were observed in the XRD and FTIR spectra before and after the reaction, confirming the regeneration of the catalyst in the cycle photocatalytic reaction. Due to the degradation of pollutants, the active O species were consumed on the surface of the material, and the gCN skeleton was reduced, as shown in Fig. 9. However, as an important component of the Z-type heterojunction, Ag/AgBr species did not decrease but continued to contribute in the photocatalytic degradation of pollutants.

### 3.4 Photoelectric property analysis

**Fig. 10:**
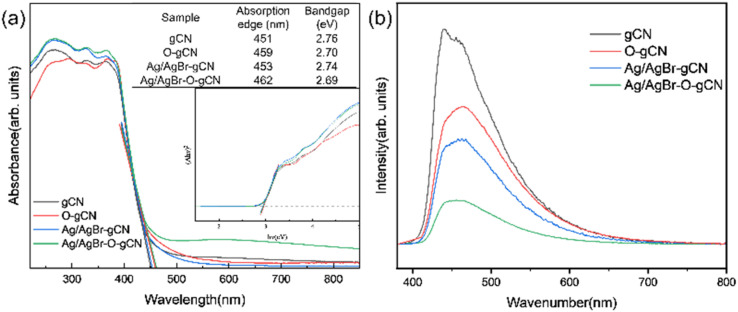
(a) UV-vis (b) PL spectrum of gCN, O-gCN, Ag/AgBr-gCN, and Ag/AgBr–O-gCN.

Fig. 10a showed the UV-vis spectra of gCN, O-gCN, Ag/AgBr-gCN, and Ag/AgBr–O-gCN, with their light absorption edges located at approximately 451 nm and 462 nm, respectively. Compared with that in gCN, a red-shift was found in the absorption band of Ag/AgBr–O-gCN, though slightly, owing to the introduction of Ag/AgBr. This led to an increase in the light absorption range of the sample. It could be seen that there was little difference in light utilization between Ag/AgBr–O-gCN and gCN, which proves the addition of Ag and AgBr did not alter the skeleton of gCN.^25^ The fluorescence spectra of gCN and the Ag/AgBr–O-gCN are shown in Fig. 10b. The PL intensity of Ag/AgBr–O-gCN is slighter than gCN, indicating that the loading of Ag/AgBr onto gCN is more beneficial to the separation of photogenerated carriers.

**Fig. 11:**
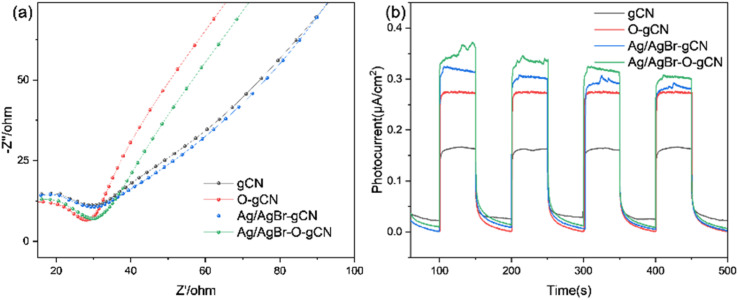
(a) EIS Nernst curves and (b) photocurrent responses of gCN, O- gCN, Ag/AgBr-gCN, and Ag/AgBr–O-gCN.

To analyse the electron migration and separation in the samples more directly, the EIS Nernst curves of each sample (Fig. 11a) and photocurrent responses excited by the xenon lamp light source (Fig. 11b) were further measured. As shown in Fig. 11a, the radius of the arc of Ag/AgBr–O-gCN series samples were smaller than that of gCN in the high-frequency region, indicating that the charge transfer resistance in Ag/AgBr–O-gCN were promoted.^27^ Moreover, the Ag/AgBr modification improved the absorption and utilization of light by gCN and excited more effective electrons under illumination, so its photocurrent response intensity (0.35 μA cm^−2^) was higher than that of gCN (0.15 μA cm^−2^). Ag was used as an electron storage pool, and a ternary heterojunction was constructed, which enhanced the separation of the photogenerated electron–hole pairs and improved the photocatalytic degradation of TMA.^25^

**Fig. 12:**
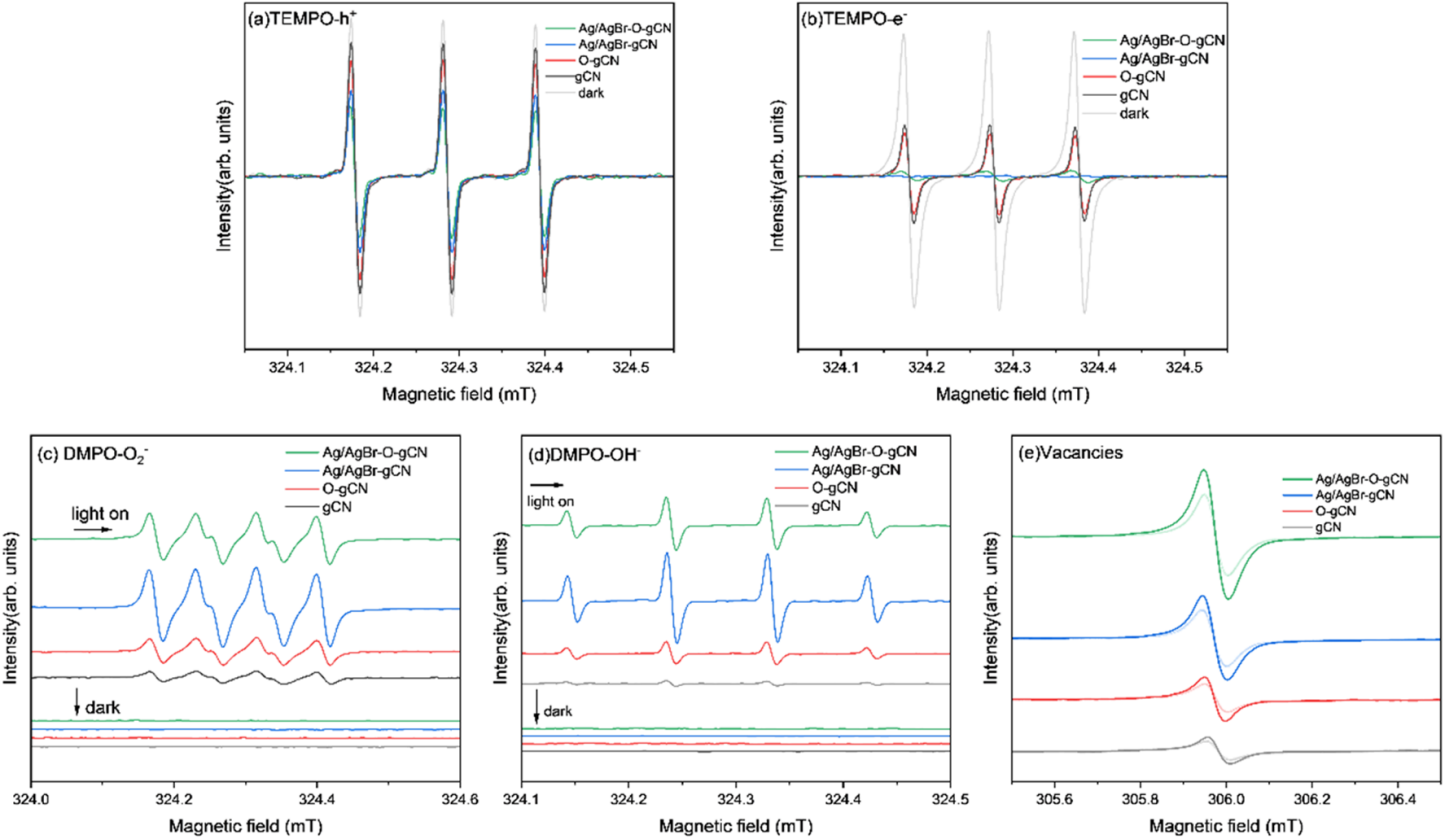
ESR spectrum of gCN, O-gCN, Ag/AgBr-gCN, Ag/AgBr–O-gCN in dark and light on for 10 min (a)TEMPO-h^+^ (b)TEMPO-e^−^ (c) DMPO ˙O_2_^−^ (d) DMPO ˙OH^−^ (e) vacancies.

Using ESR to study the effective photogenerated carriers produced by photoexcited samples more intuitively. Fig. 12a and b shows the ESR spectra of electrons and holes of each sample captured by TEMPO under dark conditions and light irradiation. It was found that when light was introduced into the system, the peak value of the sample velocity signal revealed different degrees of decline. The attenuation amplitude of the TEMPO h^+^ signal peak was Ag/AgBr–O-gCN > Ag/AgBr-gCN ≫ O-gCN > gCN, indicating that TEMPO captured more photogenerated holes in Ag/AgBr-gCN and Ag/AgBr–O-gCN. The e^−^ signal was similar to h^+^ except for the order of Ag/AgBr-gCN and Ag/AgBr–O-gCN. Ag/AgBr-gCN produced more photogenerated electrons than Ag/AgBr–O-gCN probably due to its higher content of Ag, which acted as the e^−^ storage pool and made it easier for photogenerated e^−^ to detect.^22^ Since photogenerated holes played an important role in the degradation of TMA, Ag/AgBr–O-gCN still held the best activity. The sample containing Ag/AgBr active material produced more photogenerated carriers than that of the sample without Ag/AgBr, indicating that the introduction of Ag/AgBr inhibited hole–electron recombination and increased the density of photogenerated carriers.

To further analyse the activation of H_2_O molecules in each sample, ESR analysis was performed using DMPO as a trapping agent. Fig. 12 shows the ESR patterns of ˙O_2_^−^ (Fig. 12c) and ˙OH^−^ (Fig. 12d). There was no DMPO-O_2_^−^ signal peak in the dark state for each sample, but many O_2_^−^ signal peaks were generated by the interaction of the photogenerated electrons produced by each sample with O_2_ under light. Similarly, as shown in Fig. 12d, almost no signal peaks were generated in each sample under dark conditions, and after the introduction of light, the standard quadruple peaks of hydroxyl radicals appeared (the four peaks were central, and the peak height ratio was approximately 1 : 2 : 2 : 1). These results showed that the ability of Ag/AgBr–O-gCN to oxidize and activate H_2_O molecules was much better than that of gCN, consistent with the results of the photocatalytic degradation experiment (Fig. 7). Ag/AgBr-gCN has a stronger ability to activate water molecules than Ag/AgBr–O-gCN; however, its degradation efficiency is not as good as that of Ag/AgBr–O-gCN because the sacrificial agent in methanol led to the formation of more Ag particles. Excess Ag may reduce the activity by covering the surface of gCN and AgBr, due to light shielding. Combined with the photocatalytic degradation efficiency of TMA and ESR signal results, and the precondition of dry air in the reaction tank, ˙OH^−^ and ˙O_2_^−^ play a less important role than holes in this reaction system.

The signals shown in Fig. 12e were used to evaluate the vacancies in the samples. The order of the signal intensity was Ag/AgBr–O-gCN > Ag/AgBr-gCN > O-gCN > gCN, indicating that Ag/AgBr–O-gCN was more abundant in NVs. This is consistent with the TEM-EDS results, indicating that the O heteroatom doping content was higher.

### 3.5 Analysis of energy band structure and electron transfer mechanism

**Fig. 13:**
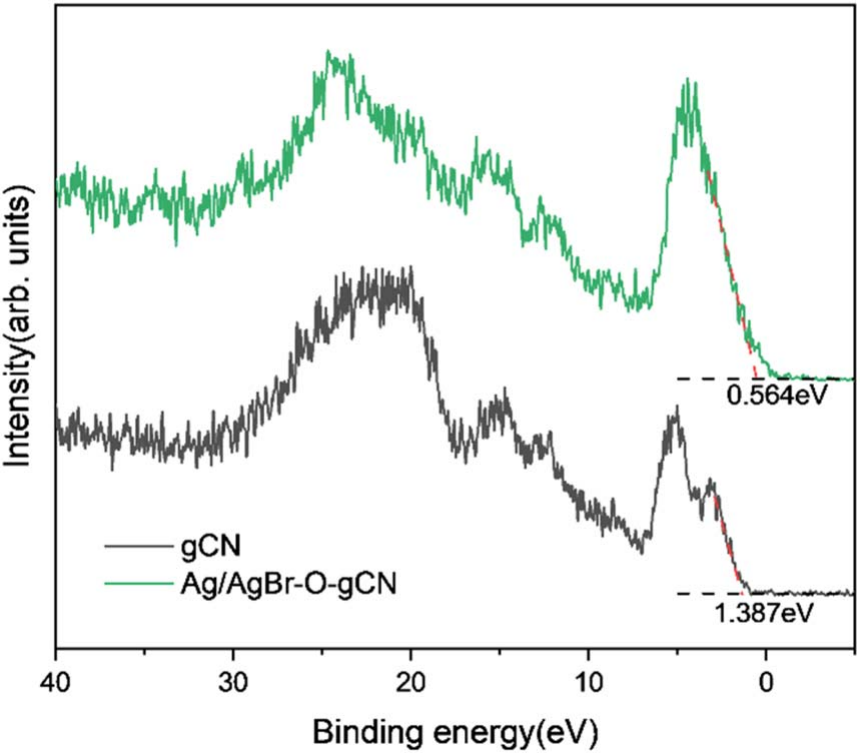
VB-XPS specter of gCN and Ag/AgBr–O-gCN.

Based on the free-radical analysis, the VB-XPS spectrum of the catalyst was further tested to reveal the band structure of Ag/AgBr-gCN. The VB of ordinary gCN was 1.387 eV, consistent with that in the literature, and the corresponding CB (*E*_CB_) could be calculated to be −1.453 eV by the equation *E*_CB_ = *E*_VB_ − *E*_g_ (Fig. 13).^27^

**Fig. 14:**
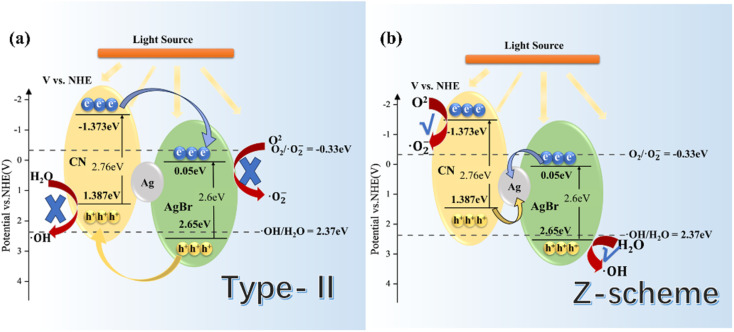
Schematic energy-level diagrams of (a) type-II and (b) Z-scheme charge transfer mechanisms for Ag/AgBr–O-gCN.

Combined with the related information on AgBr in the literature, we tried to construct a reasonable photocatalytic system.^20,21,33^ Under IPL irradiation, there is no doubt that all photocatalysts will produce photo-generated electrons and leave the same number of holes in VB. Under the type II mechanism (Fig. 14a), e^−^ accumulates on CB (0.05 eV) of AgBr and h+ accumulates on VB (1.387 eV) of gCN. However, they cannot undergo the corresponding reaction of H_2_O/˙OH^−^ (2.37 eV) and O_2_/˙O_2_^−^ (0.33 eV), resulting in the recombination of electrons and holes. In other words, the Type II mechanism was ineffective in describing the photocatalytic mechanism of Ag/AgBr–O-gCN. On the contrary, the Z-scheme mechanism was effective (Fig. 14b). The e^−^–h^+^ pairs were separated effectively when the e^−^ was transferred from the CB of gCN to Ag and recombined with h^+^ from the VB of AgBr. Consequently, the VB of gCN was enriched in holes and induced H_2_O to oxidize to ˙OH^−^ while the enrichment of electrons in the AgBr CB induced the reduction of O_2_ to ˙O_2_^−^. Here Ag acted as a Z-scheme bridge between gCN and AgBr, with the contribution of the SPR effect.

## 4 Conclusions

In this study, a ternary Z-type heterojunction photocatalyst rich in C–O bonds was constructed by the deposition of Ag/AgBr on the gCN skeleton and the simultaneous introduction of O heteroatoms by photoreduction. Ag/AgBr–O-gCN prepared with water as a sacrificial agent exhibited excellent photocatalytic degradation activity for TMA, and its kinetic constant was as high as 0.0928, which is 2.3 times that of methanol as a sacrificial agent. When methanol is absent during the photoreduction reaction, the gCN framework becomes the electron provider in the photoreduction reaction, leading to the combination of Ag/AgBr and the gCN framework to form a Z-type heterojunction. Related characterization methods indicated that Ag/AgBr–O-gCN, the catalyst with the best performance in this study, had good photo-charge separation efficiency. This was mainly attributed to the establishment of a Z-type heterojunction system with Ag nanoparticles being the recombination site, thus effectively reducing the electron–hole recombination rate. This study leads to a new approach to solve the problem of the high photogenerated charge recombination rate of g-C_3_N_4_ and supplies a new idea for the construction of ternary heterojunctions rich in oxygen heteroatoms.

## Author contributions

Xinru Chen: conceptualization, methodology, investigation, and writing; Feiyang He: methodology; Zeyu Duan: methodology; Haiqiang Wang: funding acquisition, supervision, and writing–review & editing. Zhongbiao Wu: supervision.

## Conflicts of interest

All authors disclosed no relevant relationships. The author(s) declared no potential conflicts of interest with respect to the research, authorship, and/or publication of this article.

## Supplementary Material
